# Transformers for single-cell RNA sequencing: a survey

**DOI:** 10.1093/bib/bbag332

**Published:** 2026-06-29

**Authors:** Tianxing Hu, Zhi Wei

**Affiliations:** Department of Computer Science, New Jersey Institute of Technology, Guttenberg Information Technologies Center (GITC), Suite 4100, University Heights, Newark, New Jersey 07102, USA; Department of Computer Science, New Jersey Institute of Technology, Guttenberg Information Technologies Center (GITC), Suite 4100, University Heights, Newark, New Jersey 07102, USA

**Keywords:** deep learning, self-attention, single-cell RNA sequencing, transfer learning, transformer

## Abstract

Transformers have demonstrated remarkable success in the field of deep learning, attracting significant attention from researchers and driving investigations into their applications in biomedical data analysis. Single-cell RNA sequencing (scRNA-seq) is an emerging resource important for research on disease progression and tumor microenvironments. However, scRNA-seq datasets are characterized by challenges including sparseness, high-dimensionality, large-scale, and sensitivity to batch effects. These features often necessitate substantial computational resources and can drag the performance of conventional neural networks, which frequently fail to deliver satisfactory results. The application of Transformers to single-cell sequencing is promising to solve these problems, and due to its self-attention mechanism and transfer learning paradigm, Transformers have significantly improved its model performance. This survey aims to provide a comprehensive overview of Transformers applied to scRNA-seq. It systematically analyzes the construction and capacity of Transformers in two fields: (i) Transformers designed for specific tasks, and (ii) Transformers developed to address multiple downstream tasks, often referred to as foundation models. In addition, this work examines Transformer models from the perspectives of performance, computational efficiency, interpretability, and scalability, and outlines potential avenues for future research, including emerging efforts on other omics layers beyond scRNA-seq. By offering a detailed analysis, we aim to provide a practical resource for both newcomers and experienced researchers, push forward the development of Transformer-based models for single-cell sequencing, address current challenges, and inspire further progress in the field.

## Introduction

Transformers, introduced in 2017, have revolutionized deep learning, which efficiently captures global dependencies and reduces computational cost. This survey examines the application of Transformers in single-cell sequencing data analysis. Single-cell RNA sequencing (scRNA-seq) enables transcriptomic profiling at the single-cell level for millions of cells, supporting cell classification and characterization [1]. Despite advances that have increased throughput and reduced costs, scRNA-seq data remain sparse and variable, often affected by dropout events and batch effects, highlighting the need for robust analytical methods.

Recently, the application of Transformers to scRNA-seq data has demonstrated significant progress in addressing the constraints, often surpassing the performance of other state-of-the-art methodologies. Several Transformer-based models [2–9] have significantly advanced single-cell analysis by introducing innovative strategies to capture the complexity of gene expression data. These approaches include modeling entire gene expression sequences, incorporating rank, and regulatory information, applying auto-regressive training techniques, and reconstructing various levels of sequencing depth. Additionally, progress has been made in integrating multimodal and cross-species data, combining textual metadata with gene profiles, and leveraging protein language models to improve interpretability and performance. Collectively, these developments have enhanced the scalability, accuracy, and biological relevance of Transformer-based methods in this domain.

Several prior reviews [10–12] have examined deep learning methods for single-cell transcriptomics or Transformer models in broader genomic and bioinformatics contexts; however, they either appeared prior to the recent development of Transformer-based foundation models for scRNA-seq or lack a systematic, technical comparison of model architectures, input encodings, and training strategies. A recent Nature Methods Perspective [13] provided a high-level, modality-spanning overview of Transformer applications across multiple single-cell omics, focusing primarily on conceptual adaptation, challenges, and future directions. In contrast, the present survey deliberately focuses on scRNA-seq, which currently represents the most mature and extensively explored setting for Transformer-based models, and offers a technically detailed, model-centric comparison summarized through unified tabular analyses of architectures, encoding schemes, training objectives, scalability, and downstream tasks. While this survey primarily focuses on scRNA-seq, other modalities are not reviewed in depth due to their distinct data structures and the current concentration of transformer foundation models in transcriptomic applications. Representative extensions to other omics layers are discussed as methodological generalizations, with broader implications outlined in the Discussion and Conclusion and future directions sections.

**Figure 1:**
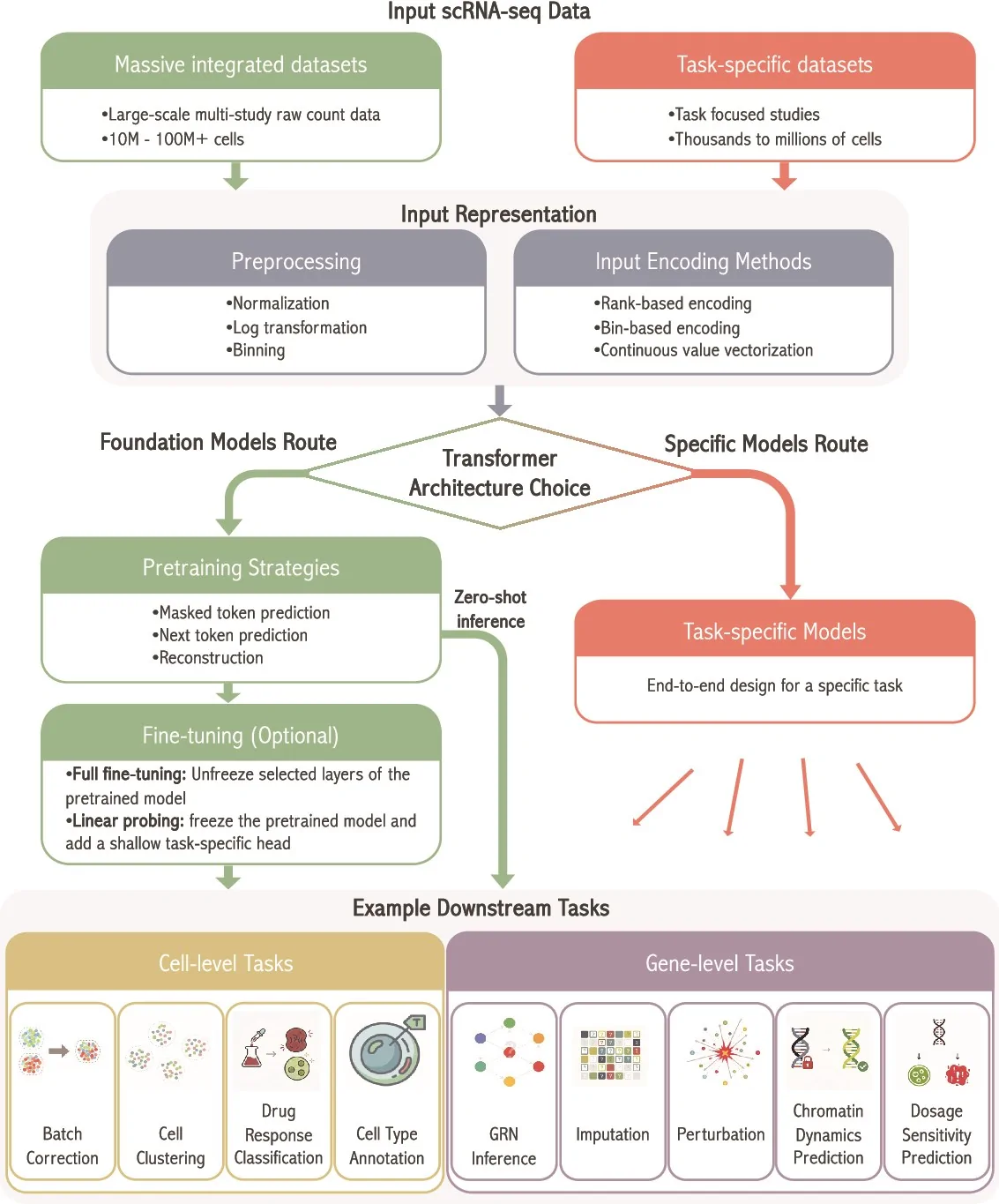
Summary illustration of Transformer models for scRNA-seq, showing that Transformer-based methods can be broadly categorized into foundation models and task-specific models.

This survey provides a comprehensive overview of Transformer applications in single-cell sequencing analysis, categorizing existing models into two types: foundation models and task-specific models (Fig. 1). Foundation models leverage unsupervised learning on large-scale data to generate gene and cell embeddings, which are fine-tuned for diverse downstream tasks. In contrast, task-specific models are tailored for defined objectives such as cell type annotation, biological network inference, imputation, and perturbation, often incorporating specialized architectural modifications. We compare foundation models across key aspects—data preprocessing, tokenization, architecture, training, fine-tuning, and downstream use (Tables 1–5)—and provide an in-depth analysis of representative models including Geneformer [3], scGPT [4], and scFoundation [5]. For task-specific models, we highlight their algorithmic innovations aligned with particular analytical goals.

**Table 1:** The scales of foundation models

Model	Data size	Max sequence length	Parameters
scBERT [2]^a^	1 126 580	16 906	—
scGPT [4]	33 m	—	—
Geneformer [3]	29 900 531	2048	—
scFoundation [5]	50 m	19 264	100 m
tGPT [14]	22.3 m	128	—
SCimilarity [15]	22.7 m	28 231	—
GeneCompass [7]	126 m	2048	100 m
Nicheformer [6]	110 m	1500	49.3 m
LangCell [8]	27.5 m	2048	—
UCE [9]	36 m	1024	650 m

$^{\mathrm{a}}$ scBERT [2] is classified as a specific model; however, it is included in this comparison due to its utilization of the transfer learning paradigm.

**Table 2:** Preprocessing and embedding techniques

Model	Norm within cells	Norm across cells	Log transformation	Binning$^{\mathrm{a}}$
scBERT [2]	x	—	x	x
scGPT [4]	x	—	x	x
Geneformer [3]	x	x	—	—
scFoundation [5]	x	—	x	—
SCimilarity [15]	x	—	x	—
GeneCompass [7]	x	x	—	—
Nicheformer [6]	x	x	—	—
LangCell [8]	x	x	—	—
UCE [9]	—	—	x	—

$^{\mathrm{a}}$ Binning represents whether discrete gene expression values are divided into bins.

**Table 3:** Training strategies of foundation models

Model	Training process
scBERT	Reconstruction^a^
scGPT	Autoregressive
Geneformer	MLM^b^
scFoundation	Reconstruction
tGPT	Autoregressive
SCimilarity	Reconstruction
GeneCompass	MLM + Reconstruction
Nicheformer	MLM
LangCell	MLM + cell–cell contrastive learning + cell-text contrastive learning + cell-text matching
UCE	Binary prediction for gene expression

$^{\mathrm{a}}$ Refers to training the model to reconstruct gene expression data.$^{\mathrm{b}}$Masked language modeling (MLM) involves predicting masked gene tokens as the objective of the training process.

**Table 4:** The training resources of foundation models

Model	Pretraining time	Resources	Speed-up method
scBERT	—	—	Performer
scGPT	—	—	FlashAttention
Geneformer	3 days	12 Nvidia V100 32GB GPUs	Dynamic and length-grouped padding; GPU Deepspeed
scFoundation	—	—	Performer decoder; mixed-precision training; distributed data parallelism; zero redundancy optimizer; checkpointing technique
tGPT	—	8 NVIDIA DGX A100 GPUs (40 GB memory each)	—
GeneCompass	9 days	8 Nvidia A800 GPUs	Deepspeed
Nicheformer	10 days	12 Nvidia A100 40 GB GPUs	—
LangCell	50 days	4 NVIDIA Tesla A100 GPUs	Momentum encoder [16]
UCE	40 days	24 A100 80 GB GPUs	—

**Table 5:** Downstream tasks of models^a^

Model	Cell-level tasks	Gene-level tasks
scBERT	Cell type annotation	
scGPT	Cell type annotation; batch correction for integrating multiple scRNA-seq datasets	Perturbation response prediction; integrative representation learning for scMultiomic data; gene regulatory network inference
Geneformer	Cell type annotation; batch integration	Gene dosage sensitivity prediction; chromatin dynamics prediction; network dynamics prediction
scFoundation	Read-depth enhancement; drug response prediction; annotating cell types	Perturbation prediction; gene regulatory network inference
tGPT	Clustering; inference of developmental lineage	Distinct features linked to cell types; clinical significance in bulk sequencing samples
SCimilarity	Outlier cells filteration and test datasets integration; cell type assignment; cell similarity search	Interpretable biological features; integrated gradients analysis on gene signatures; *in vitro* human cell models construction
GeneCompass	Cell type annotation; cell fate prediction	Gene regulatory network (GRN) inference; drug dose–response prediction; gene expression profile prediction; gene dosage sensitivity prediction; gene perturbation prediction
Nicheformer	Spatial cell type, niche, and region label prediction; neighborhood cell composition prediction; neighborhood cell density prediction	gene–gene regulatory dependency inference
LangCell	Novel cell type identification; cancer subtype classification; batch integration; cell type annotation	Pathway identification
UCE	Zero-shot dataset integration; zero-shot cell type alignment; cell type organization learning; novel cell type identification	

$^{\mathrm{a}}$ The execution of these downstream tasks in a zero-shot manner depends on the specific configurations of each model.

## Background

### Transformer

The Transformer [9] was originally proposed for sequence modeling in natural language processing (NLP). By relying entirely on self-attention mechanisms [17], rather than recurrent neural networks (RNNs) [18] or convolutional neural networks (CNNs) [19], it enables efficient parallel computation and effective modeling of long-range dependencies. The standard encoder–decoder architecture consists of stacked self-attention layers, with the decoder additionally employing cross-attention to integrate contextual information from the encoder for prediction.

An attention layer computes a weighted average of linear projections of all input elements $\mathbf{x}_{j}$ of the input set *S*. The weights, known as attention scores, are determined based on the relationships between the elements within *S*. The computation of the attention layer for a single element $\mathbf{x}_{i}^{\prime }$ in the output set $S^{\prime }=\left \{\mathbf{x}_{1}^{\prime }, \ldots , \mathbf{x}_{n}^{\prime }\right \}$ can generally be expressed as


\begin{align*} &\mathbf{x}_i^{\prime}=\sum_{j=1}^n {\mathrm{Attention}}(S)_{i, j} \times W^{\mathrm{v}} \mathbf{x}_j \\ \end{align*}

\begin{align*} & {\mathrm{Attention}}(S)_{i, j}=\operatorname{softmax}\left(\left(W^{\mathrm{k}} X\right)^{\mathrm{T}} W^{\mathrm{q}} \mathbf{x}_i / \sqrt{d_k}\right)_j\end{align*}

In the equations above, *X* represents a matrix composed of stacked input embeddings $\mathbf{x}_{i}$. The matrices $W^{N}$, $W^\rho$, and $W^{k}$ correspond to the value, query, and key projection matrices, respectively, and $d_{k}$ denotes the dimensionality of these projection matrices. The term Attention$(S)_{i, j}$ represents the attention function calculated between the query element *i* and the element *j*.

A Transformer Encoder Layer consists of an attention layer, a feed-forward network (FFN) [20], residual connections [21] and layer normalization (LayerNorm) [22], which can be expressed as follows:


\begin{align*} &H=\operatorname{LayerNorm}(\operatorname{Attention}(X)+X) \end{align*}

\begin{align*} & X^{\prime}=\operatorname{LayerNorm}(\operatorname{FFN}(H)+H)\end{align*}

Here, *X* and $X^{\prime }$ denote the input and output of a Transformer layer, respectively. Residual connections are implemented by adding the input *X* to the output of the attention module and, similarly, adding *H* to the output of the FFN.

The standard Transformer architecture has a quadratic time and memory complexity $O(L^{2})$, which limits its scalability to long input sequences such as gene expression data with 20 000 features—far exceeding typical input lengths like BERT’s 512 tokens. To address this, some single-cell Transformer models adopt Performer [23], a kernel-based attention mechanism that improves efficiency and scalability for long sequences while maintaining compact model size and training speed.

### Transfer learning

Pretraining and fine-tuning have become a pair of crucial paradigms in the Transformers. Pretraining generally refers to training models from scratch on large datasets, while fine-tuning revises slightly the pretrained model parameters, in which only a tiny amount of labeled data is needed, to adapt the different downstream works [24]. Since the pretraining model and fine-tuning model share the same model architecture, it is required to select a suitable model for both pretraining and fine-tuning, and also to find large datasets that benefit the downstream works [25]. Single-cell foundation models are mainly based on Transformer encoder blocks such as BERT [26] layer.

### Single-cell transcriptomics

scRNA-seq [27] enables high-throughput, high-resolution analysis of gene expression at the individual cell level, facilitating the classification of cells based on transcriptional profiles. Despite its power, scRNA-seq data are often sparse due to dropout events and exhibits high variability in gene expression. Advances in technology have increased throughput to hundreds of thousands of cells per experiment while reducing costs, leading to the availability of large datasets across platforms and emphasizing the need to address batch effects.

scRNA-seq data support a wide range of biomedical applications, including cell type identification, clustering, gene network inference, and drug response prediction. It has become essential in fields such as developmental biology, oncology, and immunology for uncovering cellular heterogeneity that bulk RNA-seq cannot resolve.

## Transformer models for single-cell RNA sequencing

The success of Transformer models in scRNA-seq analysis lies in their several effective key ideas. First, the self-attention mechanism allows capturing long-range dependencies between sequence elements. Second, the pretraining process efficiently learns sequence representations from the external resources with a large amount of data. Those outstanding features improve the model learning capacity for single-cell sequencing, which usually appears to be large-scale and interactively dependent inside. The application of Transformers progresses by capturing the global dependencies between genes and taking advantage of external knowledge. Furthermore, it improves the model training efficiency and parameter interpretability.

### Foundation models

In this section, we elaborate the technological details of three influential single-cell foundation models: Geneformer [3], scGPT [4] and scFoundation [5]. We analyze these models from the aspects of data collection, preprocessing, model input encoding, model architecture, pretraining, fine-tuning, downstream tasks, and performance.

#### Data collection

Table 1 summarizes the scales of foundation models. Data size refers to the size of the input dataset used for pretraining the foundation models. Max sequence length denotes the maximum length of input sequences processed by the model. Parameters represent the total number of parameters in the pretrained model.

Geneformer [3] assembled a large-scale pretraining corpus, Genecorpus-30M, consisting of 29.9 million (29 900 531) human single-cell transcriptomes sourced from various public data repositories. After quality control processing, the dataset was refined to 27 406 217 cells, which served as input for the model. Data resources utilized by Geneformer [3] include major repositories [28–41], as well as direct author correspondence.

scGPT [4] collected pretraining data from CZ CellxGene [42] platform. The CZ CellxGene platform facilitates the sharing of scRNA-seq datasets. The platform enables users to download raw count data in h5ad format as an AnnData object and supports the upload of multi-species datasets by authors, integrating these sources into a cohesive dataset. Utilizing the platform’s CELLxGENE Census API, scGPT [4] collected human normal cell transcriptomes from various tissues, including the heart, blood, brain, lung, kidney, intestine, and pancreas, resulting in a dataset of 33 million cells.

scFoundation [5] gathered pretraining data from multiple human scRNA-seq public repositories, primarily including GEO, Single Cell Portal, Human Cell Atlas, and EMBL-EBI. The scFoundation [5] dataset encompasses over 50 million single cells from a wide range of organs and tissues, derived from both healthy donors and individuals with various diseases and cancer types. All data were aligned to a standardized gene list comprising 19 264 protein-coding and common mitochondrial genes, as defined by the HUGO Gene Nomenclature Committee [43].

#### Preprocessing

Table 2 summarizes the data preprocessing procedures and encoding methods. Normalization within cells refers to scaling gene expression values within each sample, while normalization across cells scales expression values of a given gene across samples. Log transformation applies a logarithmic scale to expression values, and binning determines whether expression values are discretized before vector encoding.

Geneformer [3] adopts the loompy data format instead of h5ad to improve storage efficiency and input/output performance. Its preprocessing pipeline includes total-count normalization and log transformation, followed by normalization using the non-zero median expression of each gene across samples. This strategy downweights ubiquitously expressed housekeeping genes while emphasizing lower-expression genes that are informative for distinguishing cell states.

scGPT [4] follows standard preprocessing steps, including total-count normalization and log transformation, and further applies highly variable gene (HVG) selection to reduce feature dimensionality. This approach preserves informative expression patterns while improving computational efficiency and accommodating Transformer sequence-length constraints.

scFoundation [5] preprocesses data through normalization and log transformation. Additionally, to facilitate its specialized pretraining tasks, scFoundation [5] calculates the total read counts for the target data (used for reconstruction) and the source input data as two distinct values, T and S, respectively.

#### Model input encoding

**Figure 2:**
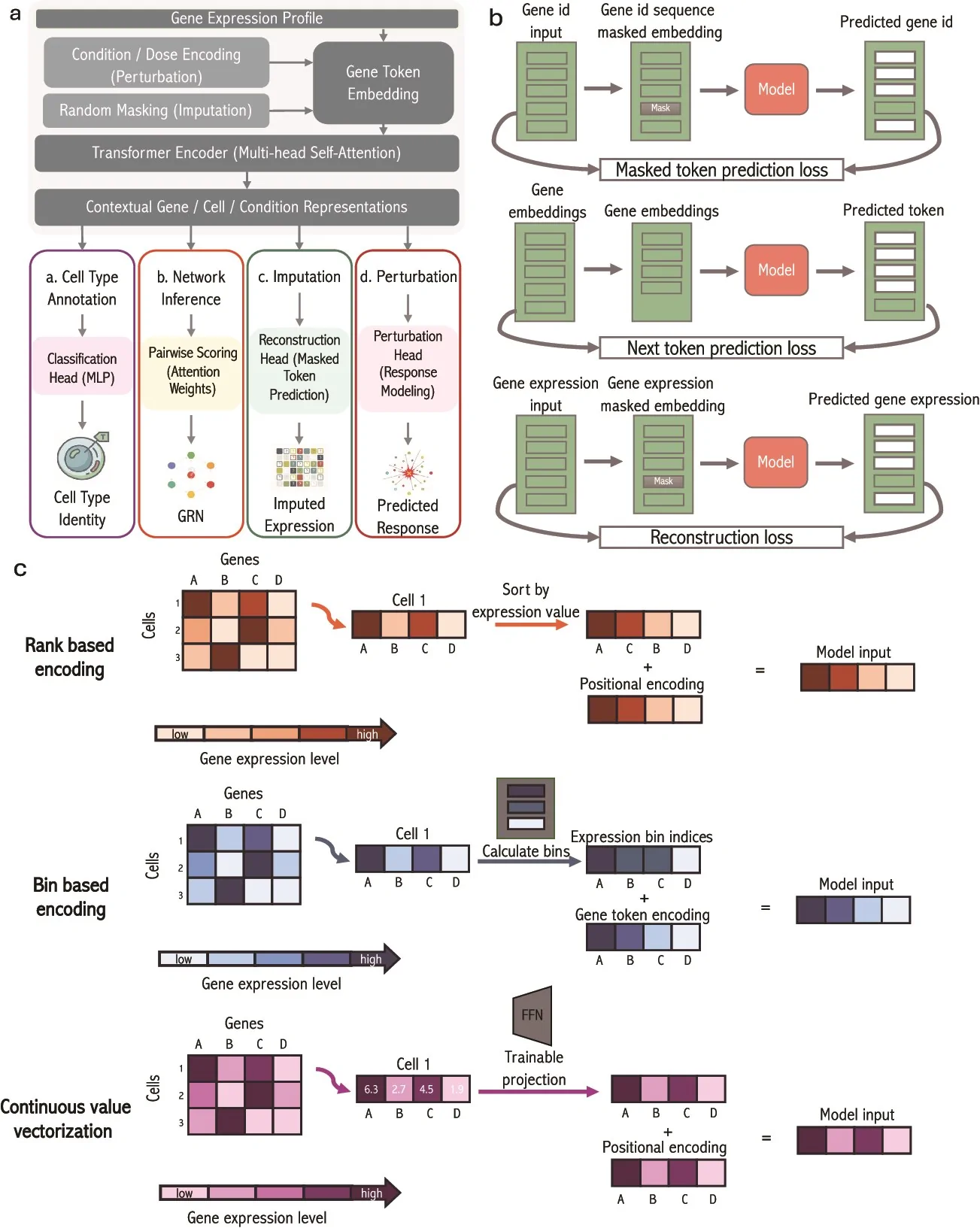
Illustration of representative transformer-based modeling strategies for scRNA-seq. (a) Task-specific transformer architectures for representative downstream tasks, including cell type annotation, biological network inference, imputation, and perturbation prediction. Input encoding modules and transformer encoders can be adapted to the task setting to generate contextual representations, which are then used by task-specific prediction heads. (b) Common pretraining objectives adopted in transformer-based foundation models, including masked token prediction, next-token prediction, and reconstruction-based learning. (c) Representative input encoding strategies used in scRNA-seq transformer models, including rank-based encoding, bin-based encoding, and continuous-value vectorization. Input encoding strategies are illustrated separately because they represent a shared design component that can be integrated into both task-specific and foundation transformer models.

The Transformer architecture, originally developed for NLP, is adapted to transcriptomic data by treating genes as tokens and cells as sequences. However, gene expression data differ fundamentally from natural language in two aspects: each gene is associated with both a gene identity and a continuous expression value that must be jointly encoded, and gene sequences lack an inherent ordering, as gene arrangement carries no semantic meaning. Consequently, the design of appropriate encoding strategies is a critical preprocessing step for adapting Transformers to gene expression data. Figure 2c illustrates three representative encoding approaches used in foundation models.

Geneformer [3] adopts a rank-based encoding strategy to jointly represent gene identity and relative expression while reducing the original dimensionality of *∼*20 000 genes. This method involves constructing a vocabulary of all detected genes in the dataset and assigning each gene a unique integer indexGenes are ranked by normalized expression within each cell, and the top 2048 gene indices are selected as input tokens. This approach preserves most expression information (93% in Geneformer-30M) while improving computational efficiency by limiting sequence length, which is critical given the quadratic complexity of Transformers.

scGPT [4] encodes continuous expression values by discretizing non-zero values into *B* bins, where each bin corresponds to an equal fraction of expressed genes within a cell. Bin edges are computed independently for each cell, allowing adaptive value quantization. Gene symbols and bin indices are embedded separately and combined element-wise to form input tokens. Additional metadata, such as batch or modality information, may also be embedded depending on the training setup.

scFoundation [5] represents expression values through continuous value vectorization. Expression values are mapped via learnable nonlinear transformations to a trainable embedding lookup table, producing value embeddings that are combined with gene name embeddings. In this formulation, value embeddings correspond to weighted combinations of embedding vectors, enabling continuous expression modeling within the Transformer framework. The gene name embeddings are retrieved from a randomly initialized lookup table, which is trainable during pretraining.

#### Model architecture

Table 3 summarizes the training strategies applied in foundation models. Reconstruction refers to training the model to reconstruct gene expression data. MLM focuses on predicting masked gene tokens. Autoregressive denotes training the model in a generative learning approach, predicting the next token in a sequence.

In terms of model architecture, Geneformer [3] is composed of 6 BERT layers with a hidden dimension of 256, followed by an MLM output layer, which serves as a classification layer for masked tokens.

scGPT [4] utilizes an architecture with 12 Transformer encoder layers, each with a hidden size of 512.

scFoundation [5] adopts an imbalanced encoder–decoder structure. The encoder consists of 12 Transformer encoder layers with a hidden size of 768, employing a vanilla Transformer block and a large parameter size. Conversely, the decoder is implemented with Performer [23] blocks, characterized by a comparatively smaller parameter size. The encoder operates on nonzero and nonmasked expressed genes as input, while the decoder processes all genes, leveraging the efficiency and scalability of the Performer [23] block.

#### Pretraining

Table 4 provides an overview of the training resources used in the pretraining of foundation models, including the duration of pretraining, the hardware computing resources employed, and the methods implemented to accelerate pretraining.

Geneformer [3] was pretrained by masking 15% of genes per transcriptome and predicting them based on unmasked context, using a custom tokenizer with dynamic, length-grouped padding to reduce computational overhead and achieve a 29.4*×* speedup. Minibatches were length-ordered and dynamically padded to minimize memory waste. Training employed the Huggingface Transformers library and DeepSpeed for distributed GPU optimization, improving memory efficiency across large-scale datasets. The model was trained with a batch size of 12 for 3 epochs, using Adam with weight decay (0.001), a peak learning rate of $1 \times 10^{-3}$, and a linear scheduler with 10 000 warmup steps to ensure stability and generalization.

During pretraining, scGPT [4] was trained on a dataset of 33 million cells, using 99.7% for training and 0.3% for validation. Only non-zero gene expressions were included, with a maximum input length of 1200 genes, randomly sampled when exceeded. The model adopted a generative next-gene prediction strategy, incorporating a dynamic attention masking mechanism to simulate the prediction of unknown genes based on known ones. The ratio of genes to generate during pretraining was uniformly sampled from three options: 0.25, 0.50, and 0.75. Pretraining was conducted with a batch size of 32 for 6 epochs, using the Adam optimizer, an initial learning rate of $1 \times 10^{-4}$, and a weight decay of 0.9 per epoch to enhance model stability and generalization.

scFoundation [5] introduced a Read-Depth-Aware pretraining strategy to address read depth variability in large-scale single-cell data. In RDA modeling, the model predicted masked gene expression values for a cell using its context genes. The context was derived from either a duplication or a low-read-depth variant of the cell’s gene expression profile. Both zero- and nonzero-expressed genes in the input sample were randomly masked. The unmasked genes and read depth indicators are utilized to predict masked gene expression. Pretraining employed the AdamW optimizer with a peak learning rate of $(1 \times 10^{-4})$, a 10 000-step warmup, a batch size of 128, and 8-step gradient accumulation to ensure efficient and stable training.

#### Fine-tuning

During fine-tuning, different models adopt distinct approaches to determine which parameters from the pretrained model are designated as trainable for specific downstream tasks.

Geneformer [3] adjusted the number of frozen layers based on the specific downstream tasks for fine-tuning. For the tasks of dosage-sensitive versus insensitive classification and chromatin dynamics long-range versus short-range predictions, four layers were frozen.

scGPT [4] maintained the same model layer configuration as the pretrained model for tasks, such as scRNA-seq batch integration, cell type annotation, and perturbation prediction, with all model parameters being learnable. However, for the task of multi-omic integration, scGPT [4] utilized frozen embeddings from the pretrained model and constructed a new model with four stacked Transformer blocks specifically for this task.

In fine-tuning, scFoundation [5] adopted a linear probing approach for tasks such as drug response prediction on bulk data, single-cell drug response classification, and single-cell perturbation prediction. This involved adding a task-specific prediction head to the output of the pretrained model without further fine-tuning the pretrained model itself. Instead, the downstream task model was trained using the cell or gene context embeddings obtained from the pretrained model. For the cell type annotation task, scFoundation [5] fine-tuned only a single encoder layer and incorporated a multi-layer perceptron head to predict labels.

#### Downstream tasks

The primary objective of single-cell foundation models is to generate cell and gene representations that enable a variety of downstream tasks in real-world applications. Based on the level of biological entities they operate on, these tasks can be categorized into cell-level tasks and gene-level tasks, which reflect fundamentally different modeling goals and sources of supervision.

Cell-level tasks focus on understanding and organizing individual cells or cell populations based on learned cell representations. These tasks aim to capture cellular identity, heterogeneity, and relationships across conditions, datasets, or biological contexts. Typical examples include cell type annotation and clustering, which assess whether a model can accurately distinguish known cell types and discover coherent cell populations. Dataset integration and batch correction further evaluate a model’s ability to align cells across experiments while preserving biological variation. More advanced cell-level applications extend to cell fate prediction, identification of novel or rare cell states, and learning higher-order organizational structures such as developmental trajectories or spatial and niche-level organization in tissues.

In contrast, gene-level tasks aim to model gene-centric mechanisms and functional relationships underlying cellular behavior. These tasks often operate on gene embeddings, attention patterns, or inferred interactions, and evaluate whether learned representations encode meaningful regulatory, functional, or causal relationships among genes. Representative gene-level applications include GRN inference and gene–gene dependency modeling, which seek to uncover regulatory interactions and coordination among genes. Other tasks, such as gene dosage sensitivity prediction, chromatin or network dynamics prediction, and perturbation or drug response prediction, assess a model’s ability to predict system-level responses to genetic or chemical perturbations. In addition, gene-level interpretability analyses, such as identifying salient gene features, pathway-level signatures, or clinically relevant biomarkers, play an important role in translating learned representations into biological insights and hypotheses.

The downstream tasks of three models—Geneformer [3], scGPT [4], and scFoundation [5]—can be categorized into cell-level and gene-level applications, highlighting their diverse capabilities. For Geneformer [3], the primary cell-level task is cell type annotation, while the gene-level tasks include gene dosage sensitivity prediction, chromatin dynamics prediction, and network dynamics prediction. The scGPT [4] model addresses cell-level tasks such as cell type annotation, cell type clustering, and batch correction, with gene-level tasks focusing on enriched pathway analysis and perturbation prediction. Finally, scFoundation [5] supports cell-level tasks, including cell clustering, cell type annotation, drug response prediction, and single-cell drug response classification, while its primary gene-level task is perturbation prediction. This categorization underscores the broad applicability of these models in both cell-level and gene-level downstream analyses.

Table 5 outlines the downstream tasks carried out by these models.

### Task-specific models

#### Cell type annotation

Cell type annotation aims to identify and label cell types or states in scRNA-seq data based on gene expression profiles. It is a fundamental task for characterizing cellular composition and functional heterogeneity in complex tissues and plays a central role in developmental biology, oncology, and immunology.

Prior to the adoption of Transformer-based models, cell type annotation primarily relied on marker gene-based approaches (e.g. SCINA [44]), correlation-based methods (e.g. Seurat [45] and SingleR [46]), and supervised or semi-supervised classifiers (e.g. scNym [47] and Scibet [48]). These approaches suffer from limitations such as sensitivity to manually curated markers, susceptibility to batch effects, or loss of high-dimensional gene interaction information during feature selection.

scBERT [2] (single-cell Bidirectional Encoder Representations from Transformers) is an early Transformer-based model for scRNA-seq cell type annotation that follows a BERT-style pretraining and fine-tuning paradigm. It discretizes continuous gene expression values into bins and treats them as tokens, enabling self-supervised masked modeling on large-scale unlabeled scRNA-seq data (PanglaoDB). To handle long-gene sequences efficiently, scBERT adopts the Performer architecture, and the pretrained model is subsequently fine-tuned for task-specific cell type annotation.

TOSICA [49] (Transformer for One-Stop Interpretable Cell-type Annotation) emphasizes interpretability through a domain-knowledge-aware embedding strategy. Genes are mapped to pathway-level tokens via a masked projection matrix, allowing attention weights to be directly traced back to biological pathways. This design enhances model interpretability while achieving strong performance on study-specific datasets and demonstrates robustness to batch effects.

T-GEM [50] (Transformer for Gene Expression Modeling) applies self-attention to unordered gene expression profiles to capture global gene–gene interactions. It focuses on the interpretability of attention patterns and their associations with biological functions and marker genes. Experiments on TCGA RNA-seq and PBMC scRNA-seq datasets show that T-GEM can identify cancer-related marker genes and pathway-level regulatory hubs.

CIForm [51] (Cell-type Identification based on Transformer) is a supervised Transformer model designed for large-scale and multi-dataset cell type annotation. Inspired by vision-based patching strategies, CIForm groups HVGs into fixed-length subvectors as input tokens, improving computational efficiency. Sinusoidal positional encoding is used to capture structural relationships within the data while mitigating batch effects across datasets.ross datasets.

#### Biological network inference

scRNA-seq data can be integrated with other single-cell multi-omics modalities, such as scATAC-seq and CITE-seq, to enable joint modeling of cellular heterogeneity and molecular regulatory mechanisms, thereby facilitating biological network inference in applications including neuroscience, cancer biology, and immuno-oncology [52].

DeepMAPS [52] (Deep learning-based Multi-omics Analysis Platform for Single-cell data) performs biological network inference by integrating multiple single-cell modalities using a heterogeneous graph Transformer (HGT). It constructs a heterogeneous graph comprising both cells and genes from raw count matrices of scRNA-seq, CITE-seq, and scRNA-ATAC-seq data. A HGT with separate autoencoders for cells and genes is then applied to jointly learn their embeddings. Multi-head attention is used to model gene–cell relationships, enabling downstream tasks such as clustering and network exploration. By incorporating attention mechanisms into a graph-based framework, DeepMAPS enhances interpretability and inference of heterogeneous biological interactions.

#### Imputation

Imputation plays a critical role in recovering missing information from sparse single-cell methylation data, which is essential for studying epigenetic regulation [53].

The CpG Transformer [53] addresses single-cell DNA CpG methylation imputation by adapting the Transformer architecture to 2D methylation matrices. Unlike most single-cell Transformer models that operate on individual cells, the CpG Transformer processes matrices with cells and CpG sites as 2D. To reduce the prohibitive computational cost of full self-attention, it employs axial attention to model row-wise and column-wise dependencies separately, and further applies sliding window self-attention to restrict local interactions. This design substantially reduces computational complexity while preserving long-range dependencies.

The model is trained using an MLM objective to predict masked CpG sites from observed context. In addition, transfer learning with the CpG Transformer improves convergence and performance in downstream imputation tasks, demonstrating its efficiency and adaptability.

#### Perturbation

TAT (Transcriptomics-to-Activity Transformer) [54] is a Transformer-based model designed to predict compound activity in various biochemical or cellular assays based on gene expression profiles observed under compound treatment. Compound activity is quantified by the pAC50 value, defined as the negative logarithm of the half-maximal activity concentration.

The model’s input consists of a sequence of gene expression profiles measured at different compound concentrations, where the sequence length corresponds to the number of concentrations, and the gene expression profiles are projected onto the model’s hidden dimension. TAT is trained in a supervised manner by minimizing the mean squared error loss between the predicted and actual pAC50 values.

## Discussion

### Evaluation

The performance of single-cell Transformer models is typically evaluated by their application to downstream tasks and real-world scenarios. For single-cell foundation models, evaluation involves multiple downstream tasks, allowing for a comprehensive comparison across various aspects, including multiple cell level and gene level. For domain-specific Transformers, their effectiveness on target tasks is assessed by comparison their performance and functionality.

**Figure 3:**
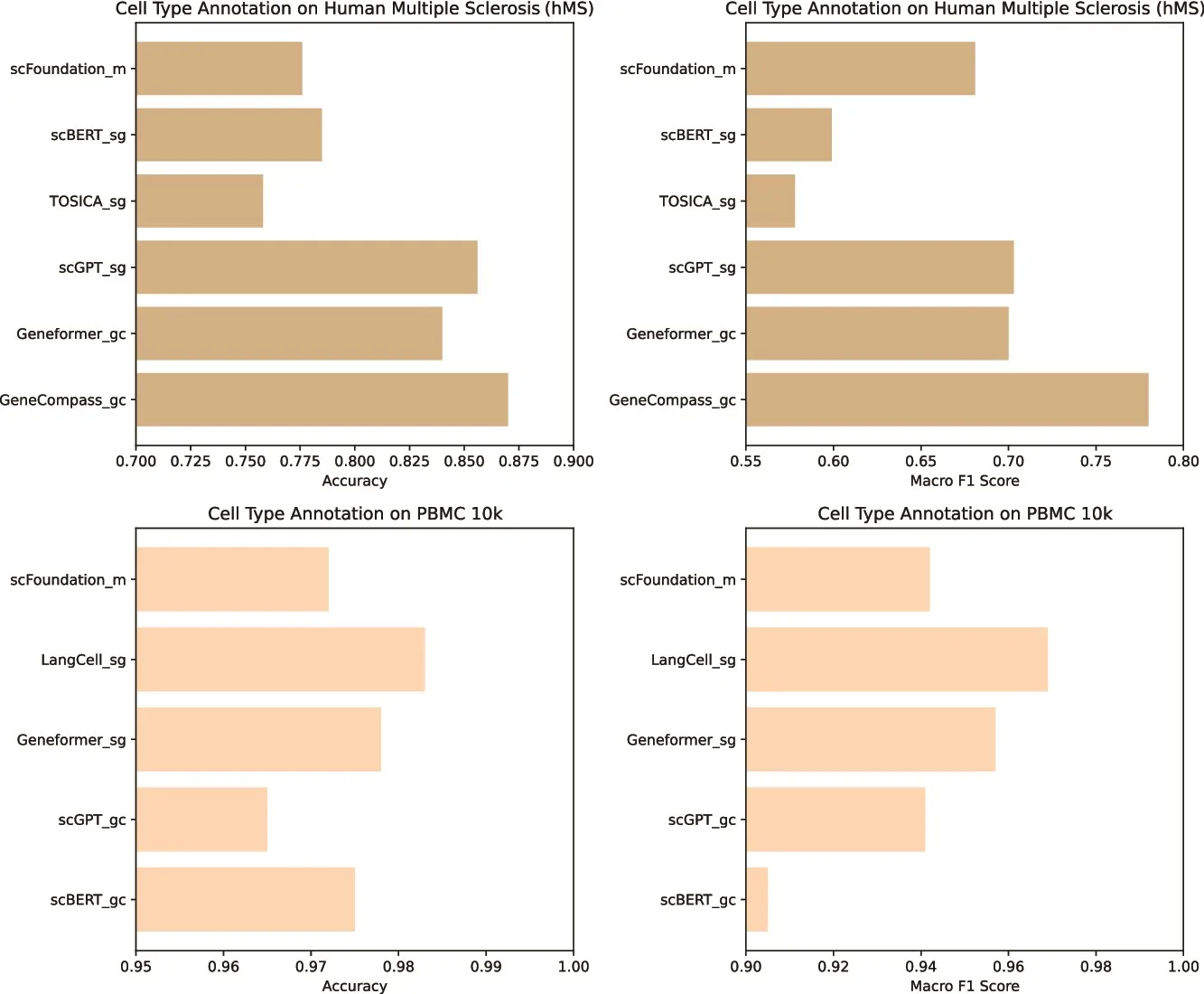
Comparison of Transformers on Cell type annotation task on human multiple sclerosis (hMS) dataset and PBMC10K dataset.

**Figure 4:**
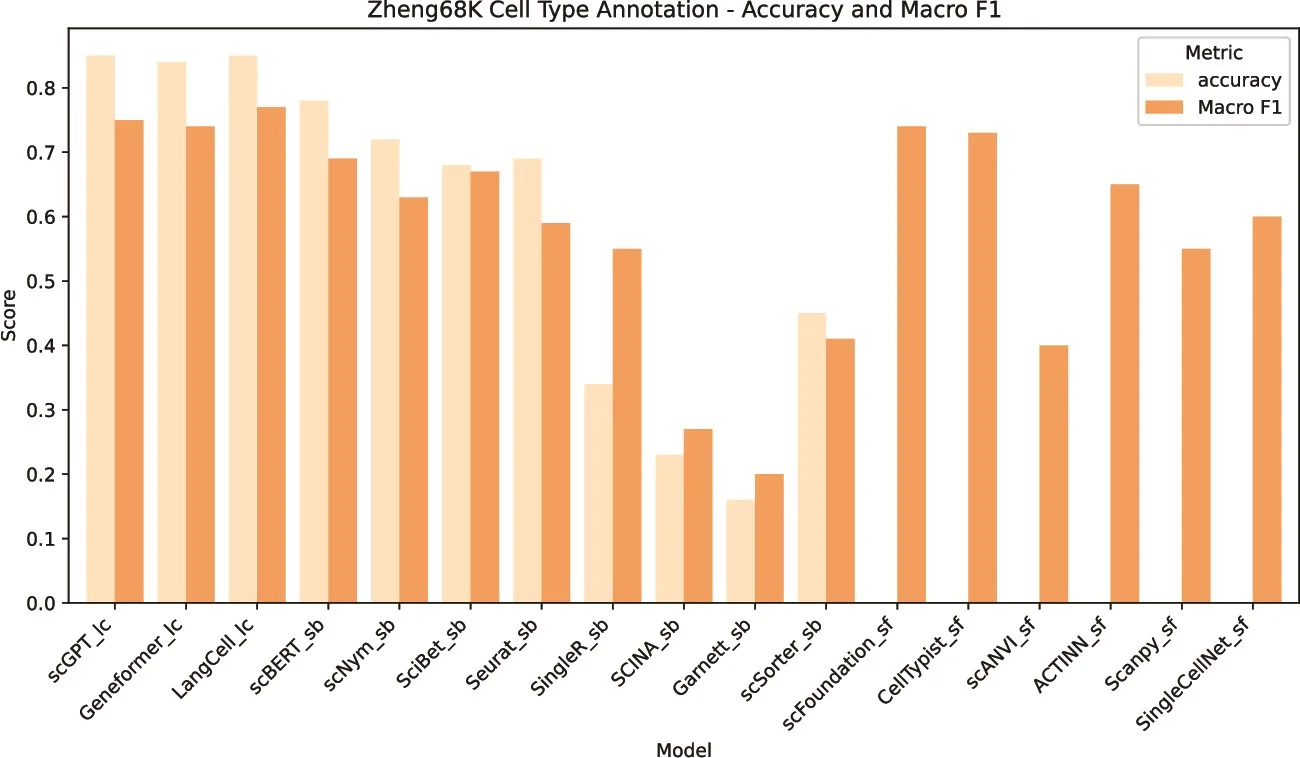
Comparison of Transformers and conventional methods on Cell type annotation task on Zheng68K dataset.

**Figure 5:**
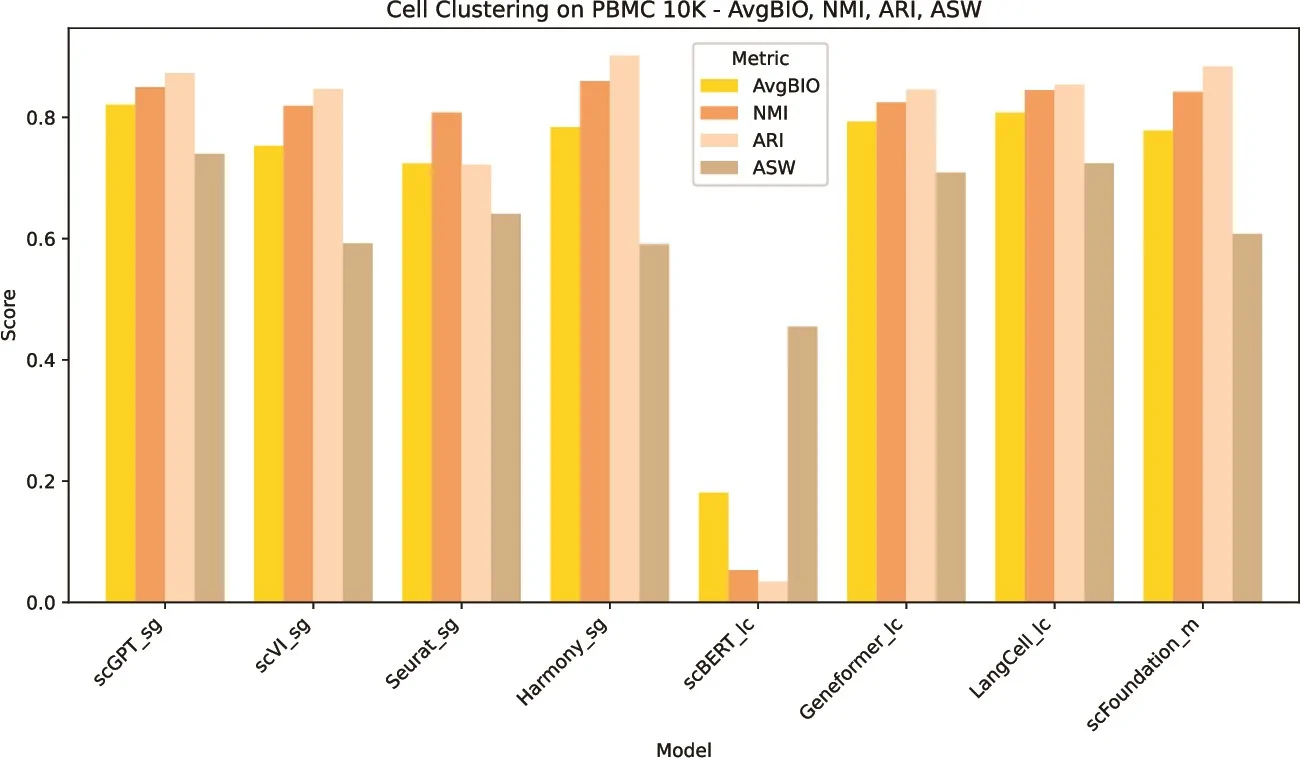
Comparison of Transformers and conventional methods on Cell clustering task on PBMC10K dataset.

**Figure 6:**
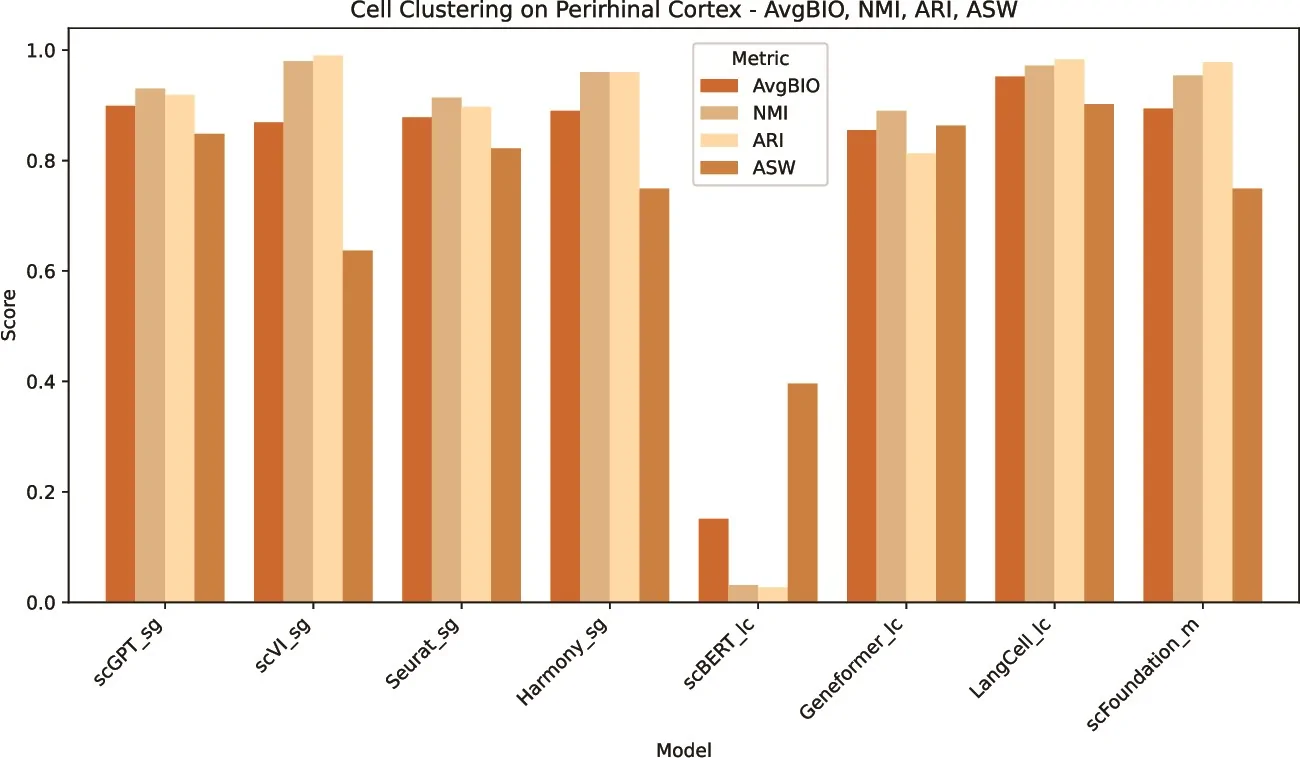
Comparison of Transformers and conventional methods on Cell clustering task on Perirhinal Cortex dataset.

Cell embeddings can be evaluated through cell-level tasks, which include cell clustering, cell type annotation, multi-omics integration, and multi-batch integration. Cell clustering is performed in an unsupervised manner by applying basic clustering methods, such as K-means, to the cell embeddings, assessing their quality. Cell type annotation involves fine-tuning the pretrained model on annotated data to perform a supervised prediction of cell types. Figure 3 demonstrates the performance of multiple Transformers on cell type annotation task. Figure 4 demonstrates the comparison among different Transformers and conventional methods over cell type annotation on Zheng68K dataset. We review results from various papers and observe that Transformers are compared against a range of conventional methods, including: scNym [47], SciBet [48], Seurat [55], SingleR [46], SCINA [44], Garnett [56], scSorter [57], CellTypist [58], scANVI [59], ACTINN [60], Scanpy [61], and SingleCellNet [62]. Figures 5 and 6 illustrate the comparison among different Transformers and conventional methods on cell clustering. Multi-omics integration and multi-batch integration evaluate the ability of cell embeddings to harmonize data from different omics and correct batch effects, respectively.

From Figs 3 to 6, we demonstrates the comparison result across different Transformers and conventional methods, on cell type annotation and cell clustering tasks. The results are reported by different papers, with _gc indicating results from GeneCompass [7], _sg indicating results from scGPT [4], _lc indicating results from LangCell [8], _sb indicating results from scBERT [2], _sf indicating results from scFoundation [5], and _m indicating results from our experiments. We can see that some Transformers demonstrate better performance than conventional models in some cases. Among Transformers, models with more parameters tend to have better performance.

Gene embeddings are evaluated through gene-level tasks such as perturbation prediction and GRN inference. Perturbation prediction involves *in-silico* simulations of gene expression perturbations to examine whether the effects on related genes or targeted cell states align with experimental results. GRN inference tests the model’s ability to infer gene regulatory relationships without prior domain knowledge provided during pretraining. Additional gene-level tasks often involve predicting gene labels in various biological contexts, requiring fine-tuning of the embeddings with labeled data.

As a downstream task, scRegNet [63] employs single-cell foundation models such as scBERT [2], Geneformer [3], and scFoundation [5] to capture context-aware gene–gene relationships in scRNA-seq data. They claim that the integration of foundation models boost the accuracy of prediction of gene regulatory link. According to their experimental results, Geneformer [3] and scFoundation [5] outperform scBERT [2] slightly in prediction, though all three models demonstrate comparable performance.

An alternative evaluation framework for single-cell foundation models parallels the scaling law paradigm in NLP. Scaling laws of neural language models [63] suggest that model performance is primarily influenced by three factors: the number of model parameters (N), dataset size (D), and compute resources (C). Performance follows a power-law relationship with each factor, provided the other two are not limiting. Single-cell foundation models also exhibit a power-law decline in validation loss with increasing model size and computational resources. For instance, scFoundation [5] recorded validation loss during pretraining of models with 3 million, 10 million, and 100 million parameters and compared their performance to scBERT [2], Geneformer [3], and scGPT [4]. The validation loss of all these models demonstrated a smooth power-law decline with increasing floating-point operations and model parameters, confirming adherence to the scaling law. Specifically, in the absence of limitations in dataset size (D) and compute (C), model performance is strongly correlated with the number of parameters (N).

Besides, Foundation models can also be assessed by evaluating the achievement of their pretraining objectives, other than the downstream tasks in domain. Common pretraining tasks include masked token prediction and next token prediction. Measuring the accuracy of models on these tasks provides a reliable metric to gauge the success of the pretraining process.

### Computational resource considerations

The Transformer architecture exhibits quadratic memory and time complexity, scaling as $O(L^{2})$ with respect to the input sequence length. Pretraining demands are substantial, depending on model size and data volume. For instance, Geneformer [3] contains six BERT layers with a hidden dimension of 256 and is pretrained on *∼*30 million data points. In comparison, scGPT [4] employs 12 Transformer encoder layers, each with a hidden size of 512, and processes *∼*33 million input data points. scFoundation [5] adopts an imbalanced encoder–decoder structure, where the encoder comprises 12 Transformer encoder layers with a hidden size of 768, while the decoder utilizes Performer [23] blocks, which are optimized for efficiency by incorporating a relatively smaller parameter size. Overall, scFoundation [5] contains over 100 million parameters and is pretrained on *∼*50 million data points. As model and dataset sizes grow, computational requirements increase accordingly. For instance, Geneformer [3] requires 3 days of pretraining using 12 NVIDIA V100 GPUs, each equipped with 32 GB of memory. Resource needs of other models vary but can be estimated relative to Geneformer [3]. Additional details are summarized in Table 4.

In contrast to the extensive resource demands of pretraining, utilizing pretrained foundation model checkpoints requires lower computational costs. However, setting up the appropriate environment for deployment and adapting the model to specific applications remain challenging. Depending on the extent of modifications required, users can employ different strategies, including inference, fine-tuning, and continual learning. For inference, memory demands remain considerable, as foundation models are substantially larger than conventional deep learning models. Loading the pretrained model and computing cell or gene representations still requires significant memory. During fine-tuning, different models suggest varying approaches, particularly regarding whether to use frozen embeddings from pretrained checkpoints. If embeddings are directly used in downstream tasks without modifying model parameters, computational costs can be greatly reduced. However, when fine-tuning involves unfreezing specific layers, both training time and memory consumption increase, as gradients can occupy more memory than the model parameters themselves. In some cases, unfreezing several or all layers may be necessary to improve downstream performance. While this incurs higher costs than inference, it remains less demanding than pretraining. Continual learning, by contrast, involves sequential training across tasks while retaining prior knowledge, which can introduce substantial computational overhead.

### Interpretability

The construction of attention layers significantly enhances the modeling of relationships between different biological entities. It allows the network to compute the dependencies between individual tokens and the rest of the tokens in a sample without the necessity of dimensionality reduction. Certain domain-specific models have been designed with a particular emphasis on biological interpretability, leveraging attention mechanisms to elucidate complex relationships within biological systems. For instance, TOSICA utilizes the Transformer architecture by maintaining traceability of all attention layers to the original input features. By integrating attention mechanisms with prior biological knowledge, TOSICA provides an advantage in interpreting attention-focused genes and pathways. Similarly, T-GEM demonstrates that attention networks in Transformer architectures can focus on specific cancer-related genes and emphasize pathways that are crucial for predicted phenotypes. The regulatory network extracted from T-GEM highlights that network hub genes are likely cancer marker genes, further supporting its interpretative capability. DeepMaps employs a HGT, which enables the estimation of gene importance in specific cell contexts, thereby facilitating discrimination of gene contributions and enhancing biological interpretability.

Beyond domain-specific models, foundation models also contribute to advancing interpretability in biological research. scGPT [4], e.g. extracts cell state-specific network activation data by aggregating single-cell signals derived from attention maps. This approach provides critical insights into context-specific gene regulatory interactions at the single-cell level, allowing for the investigation of variations across different cell states and conditions. Similarly, Geneformer [3]’s ability to predict dosage-sensitive disease genes through a context-aware *in silico* deletion strategy offers a valuable tool for interpreting genetic variants. This capability is particularly useful for prioritizing genome-wide association study hits that drive complex traits, as well as identifying the specific tissues in which these genetic variations are likely to exert their effects.

By leveraging these interpretability-enhancing mechanisms, Transformers contribute to a deeper understanding of gene regulation, disease mechanisms, and cellular dynamics.

### Scalability and efficiency

To address the substantial computational demands associated with training and fine-tuning large foundation models, various optimization techniques have been developed to enhance efficiency.

Among these, there are some typical model training tips by changing model hyperparameters to reduce computing burden such as reducing batch size and input sequence length, which serves as fundamental approaches to lowering memory consumption. More advanced techniques such as Flash Attention optimize attention computations to improve both speed and efficiency. Low-rank adaptation reduces the number of trainable parameters during fine-tuning, thereby decreasing memory requirements while maintaining model adaptability. Similarly, the Performer [23] architecture provides an alternative to conventional Transformers by approximating self-attention in a computationally efficient manner, mitigating the quadratic complexity inherent to standard attention mechanisms. Despite of model optimization, there are also some engineer techniques during model training. DeepSpeed, a specialized framework designed for large-scale training, facilitates more efficient memory usage and computational throughput, enabling the deployment of foundation models with reduced hardware constraints. Additionally, mixed-precision training further optimizes resource consumption by utilizing lower-bit floating-point representations, such as FP16 or BF16, without compromising model accuracy. The integration of these techniques has proven effective in alleviating the resource burden associated with model training, making it feasible to train and adapt large-scale models within computationally constrained environments.

From the perspective of downstream applications, users must carefully evaluate whether to develop a task-specific model tailored to a particular domain or invest in a foundation model designed to generalize across multiple tasks. Task-specific models typically offer advantages in terms of reduced computational and memory requirements, as they are optimized solely for the intended application, allowing for efficient deployment and inference. In contrast, foundation models demand significantly greater computing resources during both pretraining and adaptation but provide the benefit of broad applicability across diverse tasks. The decision between these approaches ultimately depends on the trade-offs between computational efficiency and model generalization, as well as the specific requirements of the application domain.

### Foundation models beyond single-cell RNA sequencing

Beyond scRNA-seq, transformer-based foundation models have recently been extended to other omics layers, aiming to capture complementary regulatory and spatial information, i.e. not accessible from transcriptomic profiles alone.

In single-cell epigenomics, scATAC-seq data are represented as highly sparse cell-by-region matrices with noisier signals than scRNA-seq, posing challenges for representation learning. scATAC-seq provides direct insight into transcriptional regulation by enabling inference of term frequency (TF) activity, enhancer–gene associations, and regulatory rewiring [64, 65]. A representative foundation model in this domain is EpiAgent [66], a transformer-based architecture pretrained on a manually curated large-scale scATAC-seq corpus comprising over five million cells. EpiAgent tokenizes candidate cis-regulatory elements (cCREs) and ranks them using TF-inverse document frequency transformation, with pretraining objectives designed to model cCRE accessibility patterns and reconstruct chromatin accessibility signals at the cell level. The resulting model supports a broad range of downstream applications, including unsupervised feature extraction, supervised cell-type annotation, data imputation, perturbation response prediction, reference-based integration, and simulation of cell-state transitions. Related efforts include Atacformer [67], which focuses on learning embeddings for individual cCREs, and EpiFoundation [68], which incorporates gene expression signals and peak-to-gene alignment to supervise scATAC-seq representation learning, reflecting diverse design choices for modeling epigenomic data.

Recent developments further highlight a trend toward large-scale integrative modeling of multimodal data in single-cell omics. Representative examples include SCARF [69], which extends pretraining to jointly model scRNA-seq and scATAC-seq data at scale, enabling the capture of long-range relationships within genes and regulatory elements, along with modality-specific features. EpiGePT [70] demonstrates the use of transformer-based architectures to integrate sequence-level and transcription factor activity with 3D genomic context for predicting context-specific epigenomic signals, including chromatin interactions and variant effects.

Foundation models have also begun to emerge for spatial transcriptomics, which profiles gene expression in intact tissues while preserving spatial coordinates, thereby enabling the study of tissue architecture, cellular neighborhoods, and spatially organized gene programs [71]. Modeling spatial transcriptomics introduces additional complexity due to explicit spatial dependencies and platform-specific biases. scGPT-spatial [72] extends the scGPT foundation model via continual pretraining on *∼*30 million spatial transcriptomic profiles, inheriting its tokenization scheme while introducing a mixture-of-experts decoder to capture modality-specific characteristics across Visium, Visium HD, Xenium, and MERFISH platforms. By incorporating spatially aware sampling strategies and jointly optimizing intra-spot and inter-spot gene expression prediction tasks, scGPT-spatial achieves strong performance in spatial data integration, cell-type deconvolution, and gene expression imputation. Other recent models, such as stFormer [73], which integrates spatial ligand information through cross-attention mechanisms, and SToFM [74], which jointly models tissue-scale morphology, cellular microenvironments, and gene-level expression, further illustrate emerging trends toward unified spatial representation learning.

In addition to chromatin accessibility and spatial transcriptomics, foundation models have also been explored for other omics layers, including DNA methylation. Models such as MethylGPT [75] and CpGPT [76] demonstrate that large-scale pretraining on methylome data can support robust imputation and predictive tasks such as methylation profiles imputation, chronological age prediction, and mortality risk assessment, highlighting the generalizability of foundation model paradigms across diverse omics modalities.

## Conclusion and future directions

The vanilla Transformer model, originally developed for machine translation in 2017, marked the emergence of a leading class of deep learning neural networks. This architecture has advanced state-of-the-art methodologies not only in NLP but also across various domains of fundamental scientific research. Single-cell sequencing data, a critical resource for medical and biological insights, has also benefited from the adoption of Transformer models. The multi-head attention mechanism inherent in Transformers enables the capture of long-range dependencies, effectively addressing the sparsity and high dimensionality characteristic of single-cell data. Additionally, the straightforward computation of attention scores enhances model interpretability. The pretraining and fine-tuning paradigm further optimizes the utilization of large-scale scRNA-seq data by transferring knowledge gained from extensive datasets to downstream tasks with smaller datasets.

This survey provides a comprehensive overview of Transformer models applied in single-cell sequencing data analysis, classifying them into two categories: foundation models, trained for multiple downstream tasks, and task-specific models, designed for particular applications. It systematically compares these models in terms of data processing, model architecture and training, performance, and evaluation. Additionally, it delves into the technical details of three representative foundation models: Geneformer [3], scGPT [4], and scFoundation [5]. The survey further highlights innovations in Transformer models tailored to specific tasks, including cell type annotation, biological network inference, imputation, and perturbation.

Future advancements in sequencing technologies are expected to significantly increase the volume of single-cell data, creating opportunities for large foundation models to demonstrate their capabilities. In parallel, emerging large-scale single-cell resources (e.g. scBaseCount [77], Human-scATAC-Corpus [78], and scMMO-atlas [79]) provide increasingly comprehensive multi-omics and epigenomic datasets, which are expected to further facilitate future pretraining, benchmarking, and multimodal modeling efforts. However, leveraging Transformers for such extensive datasets will necessitate substantial computational resources for model pretraining. Developing more computationally efficient architectures and training strategies is therefore an important research direction, as exemplified by recent efforts, such as GeneMamba [80]. Furthermore, many current approaches rely on architectures not explicitly designed for single-cell sequencing, limiting their ability to address unique challenges such as data sparsity, high dimensionality, and sensitivity to batch effects. Developing optimized architectures tailored to these characteristics is crucial for achieving more effective and reliable outcomes. In terms of model interpretability, the attention mechanism of Transformers provides a promising avenue to explore underlying biological regulatory processes, such as GRNs and cell communication networks.

From a longitudinal perspective, future foundation models in single-cell transcriptomics should move beyond representation learning toward mechanistic and causal interpretation. While current transformer-based models excel at annotation, data integration, and batch correction, their long-term impact will depend on linking learned representations to regulatory mechanisms, gene programs, and cell-state transitions. Integrating perturbation data offer a promising path toward causal inference and more interpretable model predictions, while uncertainty estimation may further improve robustness in novel biological contexts.

From a horizontal perspective, the rapid emergence of foundation models across other omics layers highlights an important trend toward cross-modal generalization. Epigenomic foundation models based on scATAC-seq offer complementary views of transcriptional regulation by modeling chromatin accessibility and cis-regulatory landscapes, while spatial transcriptomics models extend transfer learning to explicitly capture spatial context and cellular microenvironments. These developments suggest that future foundation models will increasingly move toward multi-omics and spatially aware architectures, capable of jointly modeling gene expression, regulatory elements, and tissue organization.

Looking ahead, an important challenge and opportunity lies in the integration of heterogeneous omics modalities within unified foundation frameworks. Bridging scRNA-seq with epigenomic, spatial, and other regulatory layers may enable more comprehensive representations of cellular identity and function. Achieving this goal will require advances in scalable pretraining strategies, modality-specific architectural designs, and evaluation benchmarks that assess both predictive performance and biological interpretability.

**Key Points**

- Transformer models show strong potential for analyzing single-cell sequencing data due to their ability to handle sparsity and high dimensionality.
- As sequencing technologies advance, larger datasets will allow for pretraining large foundation models, although significant computational resources will be needed.
- The effectiveness of pretraining and scaling laws in single-cell applications remains an open research question.
- Many current models are not tailored to the unique challenges of single-cell data, highlighting the need for specialized architectures.
- Transformers’ attention mechanisms also offer improved interpretability, enabling deeper biological insights and integration with graph- and text-based biomedical data.

## Data Availability

This article is a literature review and does not involve the generation or analysis of new data.
